# Tocilizumab potentially prevents bone loss in patients with anticitrullinated protein antibody-positive rheumatoid arthritis

**DOI:** 10.1371/journal.pone.0188454

**Published:** 2017-11-20

**Authors:** Yi-Ming Chen, Hsin-Hua Chen, Wen-Nan Huang, Tsai-Ling Liao, Jun-Peng Chen, Wen-Cheng Chao, Ching-Tsai Lin, Wei-Ting Hung, Chia-Wei Hsieh, Tsu-Yi Hsieh, Yi-Hsing Chen, Der-Yuan Chen

**Affiliations:** 1 Division of Allergy, Immunology and Rheumatology, Taichung Veterans General Hospital, Taichung, Taiwan; 2 Department of Medical Research, Taichung Veterans General Hospital, Taichung, Taiwan; 3 Faculty of Medicine, National Yang Ming University, Taipei, Taiwan; 4 Rong Hsing Research Center for Translational Medicine, National Chung Hsing University, Taichung, Taiwan; 5 Biostatistics Task Force of Taichung Veterans General Hospital, Taichung, Taiwan; 6 Division of Chest Medicine, Taichung Veterans General Hospital, Taichung, Taiwan; 7 Department of Medical Education, Taichung Veterans General Hospital, Taichung, Taiwan; 8 Ph.D. Program of Business, Feng Chia University, Taichung, Taiwan; 9 Institute of Biomedical Science, National Chung Hsing University, Taichung, Taiwan; Charles P. Darby Children's Research Institute, 173 Ashley Avenue, Charleston, SC 29425, USA, UNITED STATES

## Abstract

Rheumatoid arthritis (RA) is associated with a high risk of osteoporosis and fracture. Interleukin (IL)-6 inhibitors may suppress osteoclast activation. Anticitrullinated protein antibody (ACPA) titers are inversely associated with bone mineral density (BMD). However, the differential effect of ACPA on bone turnover marker (BTM) and BMD changes after IL-6 inhibition remains unclear. This prospective study recruited patients with active RA with inadequate response to methotrexate or biologics. BMD was measured before and after 2-year tocilizumab (TCZ) treatment. Serum osteocalcin, N-terminal propeptide of type I collagen (P1NP), and C-terminal cross-linking telopeptide of type I collagen (CTX) levels were assessed at the baseline and after treatment. We enrolled 76 patients with RA (89.5% women, age: 57.2 ± 13.3 years) receiving TCZ. The 28-joint disease activity score was negatively correlated with BMD and T-scores of the lumbar spine and bilateral femoral neck. ACPA-positive patients had lower lumbar spine and femoral neck T-scores. After 2-year TCZ treatment, CTX levels significantly decreased (0.32 ± 0.21 vs. 0.26 ± 0.17, *p* = 0.038). Femoral neck BMD increased significantly (0.71 ± 0.22 vs. 0.69 ± 0.55, *p* = 0.008). Decreased CTX levels and improved BMD were observed only in ACPA-positive patients. After treatment, femoral neck BMD significantly increased only in patients receiving a glucocorticoid dose of ≥5 mg/day. Two-year TCZ treatment reduced bone resorption and increased femoral BMD in ACPA-positive patients. The net effects of glucocorticoids and IL-6 inhibition on BMD imply that strict inflammation control might affect bone metabolism.

## Introduction

Rheumatoid arthritis (RA) is associated with increased systemic bone loss, resulting in a high risk of hip and vertebral fractures [1–3]. Concomitant glucocorticoid treatment and chronic systemic inflammation contribute to the increased risk of osteoporosis [4,5]. Tumour necrosis factor (TNF)-α and interleukin (IL)-6 are key cytokines involved in RA pathogenesis and bone complications [6]. In the past 15 years, biological therapies targeting TNF-α were associated with reduced bone destruction and reduced systemic bone loss [7]. After TNF-α inhibition, the bone formation marker N-terminal propeptide of type I procollagen (PINP) increased, whereas the bone resorption marker C-terminal crosslinking telopeptide of type I collagen (CTX) decreased [7]. However, the effects of TNF-α blockers on the incidence of fracture remain unclear. Epidemiological studies have not reported any difference in nonvertebral fractures with the use of TNF-α antagonists [8,9].

IL-6 promotes systemic bone resorption by regulating osteoclast activation and differentiation [10]. Serum IL-6 levels were negatively correlated with the T-scores of the spine and hip in RA [11]. Tocilizumab (TCZ), an IL-6 receptor inhibitor, could effectively control systemic inflammation and reduce radiographic damage [12]. CTX decreased significantly after TCZ therapy, indicating that IL-6 inhibition reduces bone resorption [13]. Moreover, TCZ was revealed to increase bone mineral density (BMD) in patients with active RA and baseline osteopenia [14]. However, a contradictory result of no change in BMD after 48 weeks of TCZ treatment was reported [15]. Therefore, the effects of TCZ treatment on BMD remain unclear.

Several independent studies have indicated an association of anticitrullinated protein antibody (ACPA) positivity in RA with radiographic progression [16, 17]. ACPA levels were also associated with CTX in patients with RA [18]. In addition, ACPA directly induces bone loss by binding to osteoclast surfaces, leading to bone resorptive activities [18]. Recent studies have also demonstrated that ACPA titers were inversely associated with BMD in early and established RA cohorts [19–21]. Rheumatoid factor (RF) and ACPA positivity could predict the therapeutic responses of rituximab and abatacept, but not of TCZ [22]. However, the effects of ACPA positivity and changes in BMD after TCZ treatment have not yet been explored.

The purpose of the current study was to investigate the differential effects of ACPAs on bone turnover markers (BTMs) and changes in BMD after 2-year TCZ treatment in patients with RA.

## Materials and methods

### Study participants

In this study, 76 patients with RA followed at Taichung Veterans General Hospital, Taiwan, between March 2013 and May 2016 were recruited. All patients fulfilled the 2010 ACR and EULAR classification criteria for RA [23]. Enrolled patients were inadequate responders to at least two combinations of an adequate dose of methotrexate (MTX)-based conventional synthetic disease-modifying antirheumatic drugs (csDMARDs), previous biological disease-modifying antirheumatic drugs (bDMARDs), or targeted synthetic disease-modifying antirheumatic drugs (tsDMARDs). This study was approved by the Ethics Committee of Clinical Research, Taichung Veterans General Hospital (CG16070A). Written informed consent was obtained from each patient according to the Declaration of Helsinki.

### Study protocol

This was a 2-year prospective observational study. All patients received 4 mg/kg of TCZ intravenously every 4 weeks in the first 3 months. If low disease activity defined by a 28-joint disease activity score (DAS28) of <3.2 was not achieved [24], a higher dose (8 mg/kg) of TCZ was administered. Patients using bDMARDs or tsDMARDs within 2 weeks (etanercept or tofacitinib), 8 weeks (adalimumab, golimumab, or abatacept), or 6 months (rituximab) were excluded from this study. In addition, patients with a history of using antiosteoporosis medication (alendronate, ibandronate, zoledronic acid, raloxifene, denosumab, and teriparatide) were excluded. All patients received a stable dose of oral glucocorticoids and csDMARDs during the study period. The use of antiosteoporosis medication was also prohibited during the study period.

### Demographic data and RA disease activity

The demographic features, concomitant csDMARDs, and serum RF immunoglobulin M (IgM) and ACPA levels of the patients were recorded at the baseline before enrolment. The RF IgM levels were measured through nephelometry (Dade Behring Inc., Newark, DE, USA, positive if ≥14 IU/mL). The ACPA levels were determined using the EliA CCP (Phadia, Nieuwegein, The Netherlands, positive if ≥7 U/mL). The serum levels of the erythrocyte sedimentation rate (ESR), C-reactive protein (CRP), and DAS28 [25] were used to assess RA disease activity.

### BMD measurements

BMD measurements of the lumbar spine (L1–L4) and bilateral femoral neck were performed at the baseline and at the end of the study by using dual-energy X-ray absorptiometry (Lunar Prodigy, General Electric, Fairfield, CT, USA). BMD was calculated as the bone mineral content divided by the surface of the projected bone area (g/cm^2^). The least significant detectable difference was ±0.010 g/cm^2^ for the lumbar spine L1–L4 and ±0.012 g/cm^2^ for the femoral neck. T-scores were determined according to manufacturer’s reference data.

### BTM assessment

The serum levels of bone markers were examined at the baseline and the end of the study. CTX (ECLIA Modular Roche Diagnostics, Mannheim, Germany) was used as the bone resorption marker. P1NP and osteocalcin (ECLIA Modular Roche Diagnostics, Mannheim, Germany) were used as bone formation markers. Blood samples were collected in the morning with overnight fasting to avoid the effect of circadian variation on BTM.

### Statistical analysis

The demographic data are presented as the mean ± standard deviation for continuous variables and as the number (percentage) of patients for categorical variables. Parameters were examined for normality by Kolmogorov-Smirnov test. Lumbar spine, femoral neck BMD and T-scores were not normally distributed. Therefore, the correlations between RA disease activity, BTM, and BMD were examined using Spearman’s rank correlation coefficient. The Mann–Whitney U test and chi-square test were used for comparisons between continuous and categorical variables in ACPA-positive and ACPA-negative patients. Comparisons before and after TCZ treatment were performed using the Wilcoxon signed rank test. A generalized estimating equation was used to evaluate differences in bone markers and bone density between ACPA-positive and ACPA-negative patients and between patients using a daily glucocorticoid dose of ≥5 mg and those using a dose of <5 mg. All data were analysed using the Statistical Package for Social Sciences (SPSS), version 22.0. A *p* value of <0.05 was considered significant.

## Results

### Correlations between RA disease activity, BTM, and BMD

A total of 76 patients with RA (women, 89.5%; age, 57.2 ± 13.3 years) receiving TCZ were enrolled. Scatter plots of the raw data (S1 Dataset) were shown in the S1 Fig. According to Spearman’s rank correlation coefficient, the baseline DAS28 was negatively correlated with the BMD (*r* = −0.262, *p* = 0.025) and T-score (*r* = −0.271, *p* = 0.036) of the lumbar spine. The DAS28 was also inversely correlated with the BMD of the right femoral neck (*r* = −0.273, *p* = 0.023) and left femoral neck (*r* = −0.275, *p* = 0.021) and the T-score (*r* = −0.291, *p* = 0.027).

### Comparison of demographic data, BTM, and BMD between ACPA-positive and ACPA-negative patients with RA

The percentage of women (94.4% vs. 77.3%, *p* = 0.041), the ESR level (27.4 ± 34.3 vs. 11.8 ± 18.2 mm/first h, *p* = 0.05), and the RF IgM titer level (329.0 ± 771.4 vs. 65.5 ± 118.4, *p* < 0.001) were higher in ACPA-positive patients than in ACPA-negative patients (Table 1). The serum levels of osteocalcin, P1NP, and CTX at the baseline did not differ between the two groups. ACPA-positive patients had significantly lower T-scores of the lumbar spine (−0.99 ± 1.57 vs. −0.17 ± 1.30, *p* = 0.027), right femoral neck (−1.76 ± 1.03 vs. −0.98 ± 1.29, *p* = 0.043), and left femoral neck (−1.76 ± 1.44 vs. −0.88 ± 1.23, *p* = 0.036) than did ACPA-negative patients. Moreover, the BMD of the right femoral neck was lower in ACPA-positive patients (0.67 ± 0.56 vs. 0.81 ± 0.14, *p* = 0.046).

**Table 1:** Comparison of demographic data, bone turnover markers, and bone mineral density between patients with ACPA-positive RA and those with ACPA-negative RA.

	ACPA-positive (n = 54)	ACPA-negative (n = 22)	p value
Age	58.30 ± 11.42	51.36 ± 16.25	0.114
Gender†			0.041*
Male	3 (5.6%)	5 (22.7%)	
Female	51 (94.4%)	17 (77.3%)	
Disease duration (years)	9.7 ± 2.9	10.1 ± 3.7	0.739
DAS28 at baseline	4.42 ± 1.38	4.2 ± 1.48	0.628
ESR (mm/hr)	27.4 ± 34.3	11.8 ± 18.2	0.050
CRP (mg/dl)	0.71 ± 1.64	0.54 ± 1.93	0.248
RF IgM (IU/ml)	329.0 ± 771.4	66.5 ± 118.4	<0.001**
Glucocorticoid (mg/day)	5.5 ± 3.2	7.4 ± 5.1	0.169
Methotrexate (mg/week)	5.9 ± 6.2	7.8 ± 5.6	0.214
Osteocalcin (ng/ml)	16.08 ± 7.77	13.47 ± 7.49	0.353
P1NP (ng/ml)	52.65 ± 21.43	46.33 ± 21.53	0.353
CTX (ng/ml)	0.33 ± 0.21	0.22 ± 0.11	0.124
Lumbar spine			
BMD (g/cm2)	0.93 ± 0.64	1.08 ± 0.16	0.087
T-score	-0.99 ± 1.57	-0.17 ± 1.30	0.027*
Femoral neck, right			
BMD (g/cm2)	0.67 ± 0.56	0.81 ± 0.14	0.046*
T-score	-1.76 ± 1.03	-0.98 ± 1.29	0.043*
Femoral neck, left			
BMD (g/cm2)	0.66 ± 0.59	0.82 ± 0.14	0.064
T-score	-1.76 ± 1.44	-0.88 ± 1.23	0.036*

Mann–Whitney U test.

^†^Chi-square test.

*p < 0.05,

**p < 0.01

Data are presented as the mean ± standard deviation or n (%). ACPA: anticitrullinated protein antibody; BMD: bone mineral density; CRP: C-reactive protein; CTX: C-terminal cross-linking telopeptide of type I collagen; DAS28: 28-joint disease activity score; ESR: erythrocyte sedimentation rate; P1NP: N-terminal propeptide of type I collagen; RF: rheumatoid factor.

### Changes in BTM and BMD before and after TCZ treatment

After 2-year TCZ treatment, the level of the bone resorption marker CTX decreased significantly (0.32 ± 0.21 vs. 0.26 ± 0.17, *p* = 0.038, Table 2) in all enrolled patients. In parallel, the BMD of the right and left femoral neck increased after IL-6 inhibition (right: 0.74 ± 0.14 vs. 0.70 ± 0.51, *p* = 0.020; left: 0.71 ± 0.22 vs. 0.69 ± 0.55, *p* = 0.008, respectively). The T-scores of the bilateral femoral neck also improved significantly (right: −1.42 ± 1.21 vs. −1.59 ± 1.17, *p* = 0.007; left: −1.43 ± 1.40 vs. −1.54 ± 1.43, *p* = 0.018). The levels of the bone formation markers osteocalcin and P1NP did not change significantly after TCZ treatment.

**Table 2:** Bone turnover markers and bone mineral density before and after tocilizumab treatment.

	Before Tocilizumab	After Tocilizumab	p value
Osteocalcin (ng/ml)	17.57 ± 8.58	16.45 ± 6.69	0.600
P1NP (ng/ml)	50.45 ± 20.63	58.48 ± 39.18	0.599
CTX (ng/ml)	0.32 ± 0.21	0.26 ± 0.17	0.038*
Lumbar spine			
BMD (g/cm2)	0.94 ± 0.58	1.37 ± 3.17	0.238
T-score	-0.84 ± 1.56	-0.78 ± 1.50	0.637
Femoral neck, right			
BMD (g/cm2)	0.70 ± 0.51	0.74 ± 0.14	0.020*
T-score	-1.59 ± 1.17	-1.42 ± 1.21	0.007**
Femoral neck, left			
BMD (g/cm2)	0.69± 0.55	0.71 ± 0.22	0.008**
T-score	-1.54 ± 1.43	-1.43 ± 1.40	0.018*

Wilcoxon signed rank.

*p < 0.05,

**p < 0.01

BMD: bone mineral density; CRP: C-reactive protein; CTX: C-terminal cross-linking telopeptide of type I collagen; P1NP: N-terminal propeptide of type I collagen.

### Differential effects of ACPA on changes in BTM and BMD after TCZ treatment

**Fig 1:**
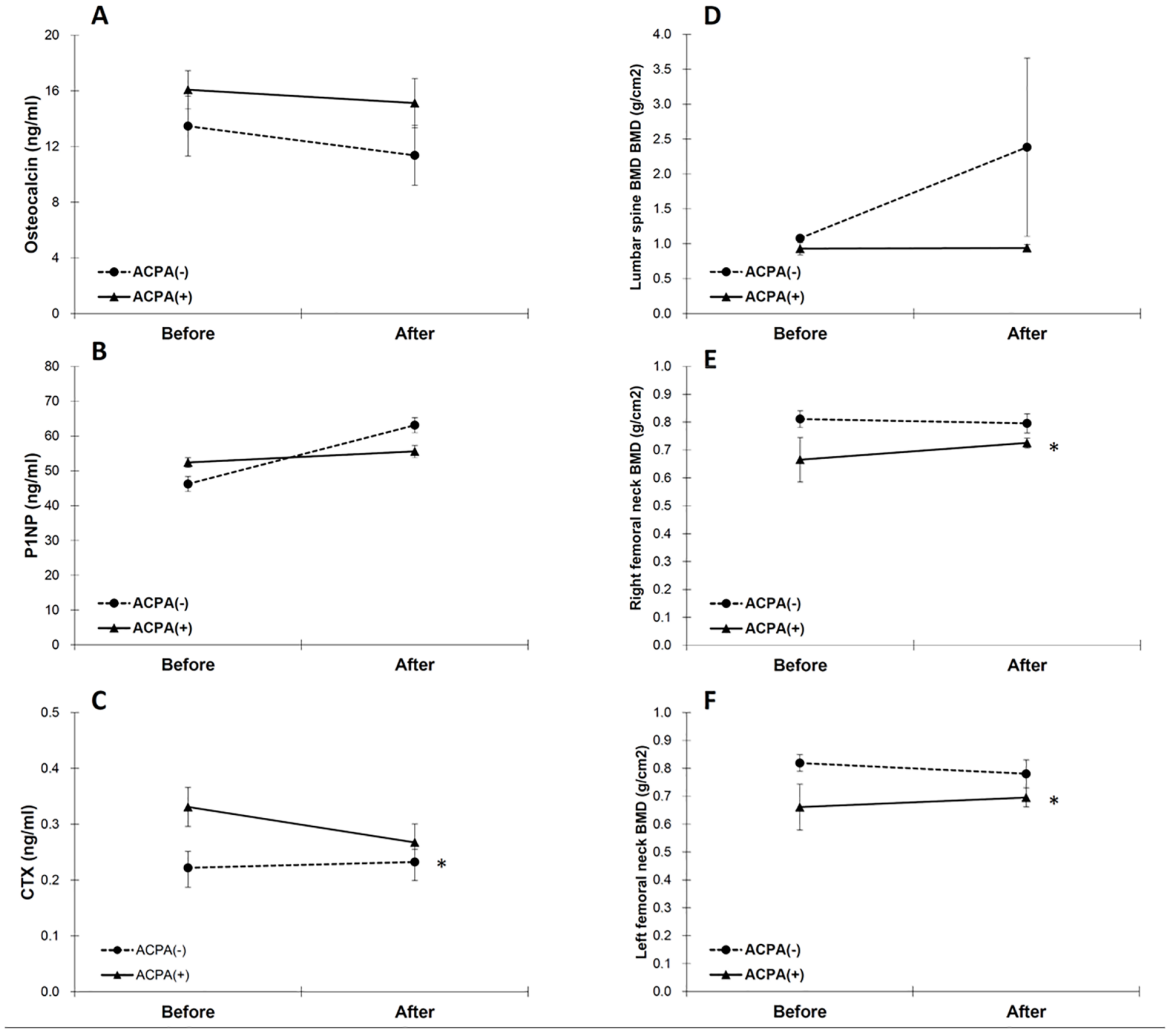
Comparison of BTM and BMD between patients with ACPA-positive RA and those with ACPA-negative RA before and after tocilizumab treatment. (A) osteocalcin, (B) P1NP, (C) CTX, (D) lumbar spine BMD, (E) right femoral neck BMD, and (F) left femoral neck BMD Error bars: mean ± standard error, *: *p* value < 0.05 by the Wilcoxon signed rank test. ACPA: anticitrullinated protein antibody; BMD: bone mineral density; BTM: bone turnover markers; CTX: C-terminal cross-linking telopeptide of type I collagen; P1NP: N-terminal propeptide of type I collagen; RA: rheumatoid arthritis.

To investigate whether changes in BTM and BMD after TCZ treatment differ with ACPA seropositivity, we further categorized patients with RA into ACPA-positive and ACPA-negative subgroups (Table 3 and Fig 1). We found a significant reduction of serum CTX levels only in ACPA-positive patients (0.37 ± 0.22 vs. 0.29 ± 0.19, *p* = 0.015, Fig 1C). Moreover, the BMD of the femoral neck improved only in ACPA-positive patients (right: 0.65 ± 0.62 vs. 0.72 ± 0.12, *p* = 0.029; left: 0.64 ± 0.66 vs. 0.69 ± 0.22, *p* = 0.020, Fig 1E and 1F). The changes in BTM and BMD after TCZ treatment did not differ significantly between ACPA-positive and ACPA-negative patients, according to the generalized estimating equation (*p* value not shown).

**Table 3:** Comparison of bone turnover markers and bone mineral density between patients with ACPA-positive RA and those with ACPA-negative RA after tocilizumab treatment.

	Before Tocilizumab	After Tocilizumab	p value
Osteocalcin (ng/ml)			
ACPA(-)	19.09 ± 9.22	17.22 ± 5.43	0.465
ACPA(+)	19.12 ± 8.25	18.17 ± 5.38	0.893
P1NP (ng/ml)			
ACPA(-)	42.51 ± 17.29	62.88 ± 38.48	0.136
ACPA(+)	53.76 ± 21.61	57.22 ± 40.58	0.705
CTX (ng/ml)			
ACPA(-)	0.21 ± 0.12	0.22 ± 0.08	0.754
ACPA(+)	0.37 ± 0.22	0.29 ± 0.19	0.015*
Lumbar spine BMD (g/cm2)			
ACPA(-)	1.08 ± 0.17	2.38 ± 5.72	0.796
ACPA(+)	0.89 ± 0.71	0.94 ± 0.33	0.183
Femoral neck, R't BMD (g/cm2)			
ACPA(-)	0.82 ± 0.15	0.80 ± 0.15	0.334
ACPA(+)	0.65 ± 0.62	0.72 ± 0.12	0.029*
Femoral neck, L't BMD (g/cm2)			
ACPA(-)	0.83 ± 0.14	0.78 ± 0.22	0.427
ACPA(+)	0.64 ± 0.66	0.69 ± 0.22	0.020*

Wilcoxon signed rank.

*p < 0.05

ACPA: anticitrullinated protein antibody; BMD: bone mineral density; CTX: C-terminal cross-linking telopeptide of type I collagen; P1NP: N-terminal propeptide of type I collagen; RA: rheumatoid arthritis.

### Differential effects of a glucocorticoid dose on changes occurring in BTM and BMD after TCZ treatment

**Fig 2:**
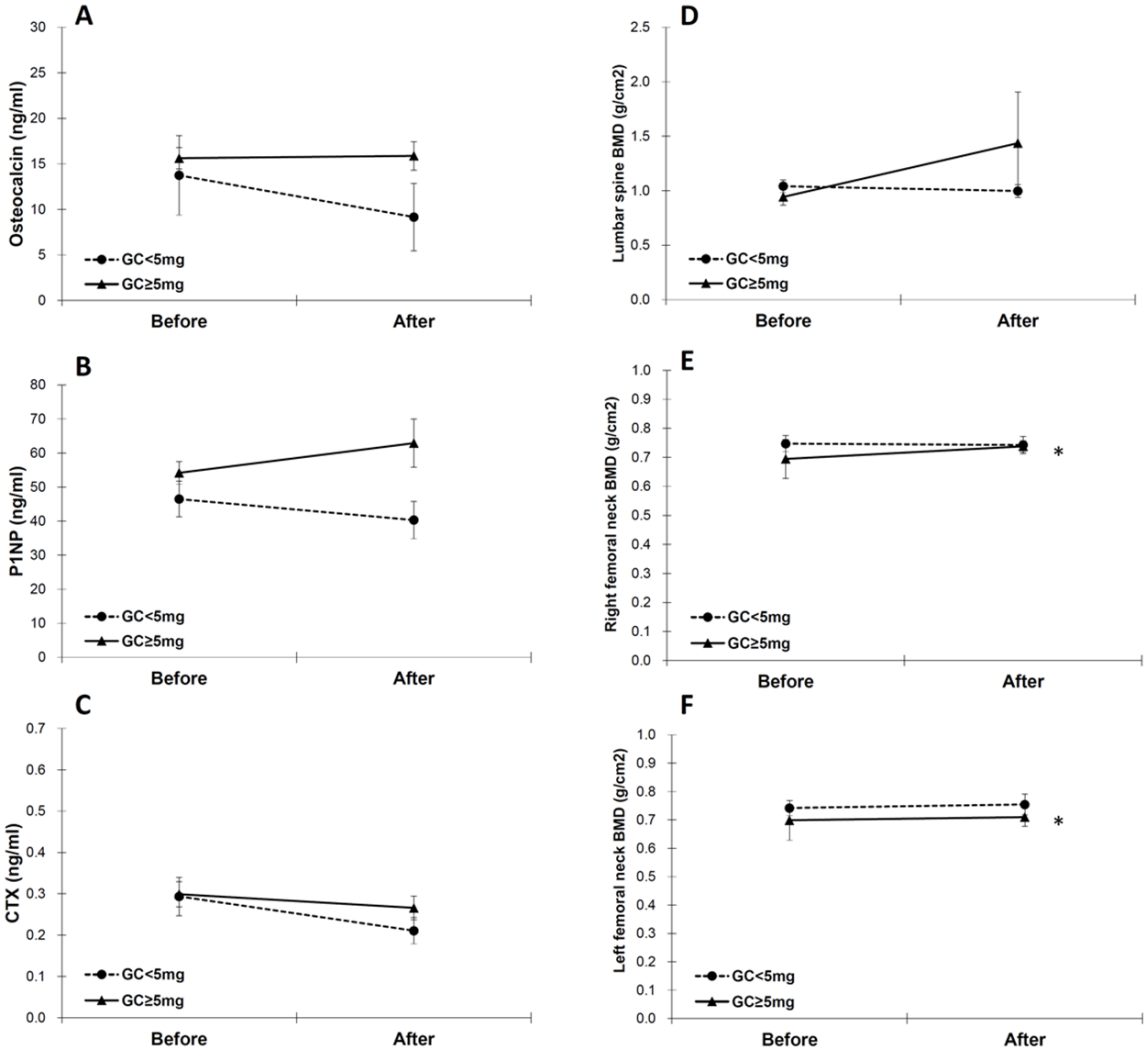
Comparison of BTM and BMD between patients with RA receiving a daily glucocorticoid dose of ≥5 mg and those receiving a daily glucocorticoid dose of <5 mg before and after tocilizumab treatment. (A) osteocalcin, (B) P1NP, (C) CTX, (D) lumbar spine BMD, (E) right femoral neck BMD, and (F) left femoral neck BMD. Error bars: mean ± standard error, *: *p* value < 0.05 by Wilcoxon signed rank test. BMD: bone mineral density; BTM: bone turnover markers; CTX: C-terminal cross-linking telopeptide of type I collagen; GC: glucocorticoid; P1NP: N-terminal propeptide of type I collagen; RA: rheumatoid arthritis.

Because BTM and BMD may respond differently according to daily glucocorticoid usage, we divided our enrolled patients into two subgroups: those who received a daily glucocorticoid dose of ≥5 mg and those who received a daily glucocorticoid dose of <5 mg (Table 4 and Fig 2). After 2-year TCZ treatment, the BMD of the femoral neck increased significantly only in patients who received a daily glucocorticoid dose of ≥5mg (right: 0.68 ± 0.56 vs. 0.74 ± 0.14, *p* = 0.034; left: 0.68 ± 0.59 vs. 0.71 ± 0.24, *p* = 0.004, Fig 2E and 2F). The changes in BTM and BMD after TCZ treatment did not differ significantly between patients who received different doses of glucocorticoid, according to the generalized estimating equation (*p* value not provided).

**Table 4:** Comparison of bone turnover markers and bone mineral density between patients receiving a daily glucocorticoid dose of ≥5 mg versus those receiving a daily glucocorticoid dose of <5 mg after tocilizumab treatment.

	Before Tocilizumab	After Tocilizumab	p value
Osteocalcin (ng/ml)			
GC<5mg	23.40 ± 12.23	18.81 ± 5.74	0.317
GC≥5mg	15.24 ± 7.02	15.50 ± 7.42	0.893
P1NP (ng/ml)			
GC<5mg	44.41 ± 19.08	39.93 ± 18.75	0.445
GC≥5mg	52.34 ± 21.01	64.28 ± 42.22	0.308
CTX (ng/ml)			
GC<5mg	0.30 ± 0.20	0.22 ± 0.12	0.114
GC≥5mg	0.32 ± 0.21	0.28 ± 0.18	0.142
Lumbar spine BMD (g/cm2)			
GC<5mg	1.01 ± 0.21	1.00 ± 0.20	0.799
GC≥5mg	0.93 ± 0.63	1.44 ± 3.45	0.212
Femoral neck, R't BMD (g/cm2)			
GC<5mg	0.76 ± 0.12	0.75 ± 0.11	0.310
GC≥5mg	0.68 ± 0.56	0.74 ± 0.14	0.034*
Femoral neck, L't BMD (g/cm2)			
GC<5mg	0.75 ± 0.11	0.76 ± 0.12	0.917
GC≥5mg	0.68 ± 0.59	0.71 ± 0.24	0.004**

Wilcoxon signed rank.

*p < 0.05,

**p < 0.01

BMD: bone mineral density; CTX: C-terminal cross-linking telopeptide of type I collagen; GC: glucocorticoid; P1NP: N-terminal propeptide of type I collagen; RA: rheumatoid arthritis.

## Discussion

The results of this study indicate that patients with ACPA-positive RA and high disease activity had a higher risk of low BMD at the lumbar spine and femoral neck than did patients with ACPA-negative RA. After 2-year TCZ treatment, bone resorption was prevented by IL-6 inhibition. We observed that the BMD of the bilateral femoral neck increased significantly. To the best of our knowledge, this is the first study to demonstrate that ACPAs might play a role in inflammation-related bone loss. Although systemic glucocorticoids may have a deleterious effect on BMD, we observed a potential benefit of IL-6 blockage in patients with RA who received a higher dose of steroids for disease management.

IL-6 receptor blockade with TCZ has been demonstrated to induce a positive balance of bone markers by increasing the P1NP level and reducing the CTX level in patients with established RA who had inadequate responses to MTX or TNF inhibitors [13, 26]. This beneficial effect is in concordance with the slow radiographic progression observed on X-ray plain films or micro-computed tomography of small hand joints [27, 28]. However, these data were mainly generated from short-term clinical trials, ranging from 16 to 52 weeks. Our results show changes in BTM after a prolonged TCZ treatment duration, suggesting that a persistent reduction in the level of the bone resorption marker CTX could still be observed after IL-6 inhibition for up to 2 years.

In arthritis animal models, anti-IL-6 receptor monoclonal antibodies prevent systemic bone loss by significantly reducing the number of osteoclast precursors in bone marrow [29]. Kume et al. reported that in a subgroup of patients with active RA and baseline osteopenia, the BMD of the lumbar spine and femoral neck increased significantly after TCZ treatment [14]. Concomitant steroid treatment was prohibited in this study. However, the beneficial effects of increased BMD by TCZ treatment were not demonstrated in the Torpedo study, which enrolled a larger sample but allowed glucocorticoid treatment [15]. The duration of TCZ treatment in both trials was 52 weeks. Our study is the first to assess BMD after 2 years of TCZ treatment. The increased BMD of the femoral neck in the present study implies that TCZ may take a longer time to prevent bone loss. In the two aforementioned studies, the DAS28 (>5.1) of enrolled participants indicated high disease activity [14, 15], which was much severe than that in our study (mean DAS28: 4.4). However, our patients had a longer disease duration and lower baseline T-scores. In a previous study, the disease duration was inversely associated with BMD in female patients with RA [30]. Disease activity predicts bone loss in early RA [31]. Moreover, the risk of osteoporotic fractures was positively correlated with the DAS28 [32]. Differences in patients’ characteristics may also contribute to the discrepancy in the study results. However, we believe that prolonged IL-6 blockade can improve bone density in patients with RA.

ACPA positivity is a well-known poor prognostic factor for erosive disease in RA [16,17]. A previous study showed that human osteoclasts expressed peptidylarginine deiminase activity eliciting protein citrullination [18]. Moreover, adoptive transfer of human ACPAs into mice induced osteoclastogenesis through inducible TNF-α release [18]. Our results are in concordance with those of recent studies indicating a negative effect of ACPAs on BMD in patients with early and established RA [19–21]. In patients with RA who received adalimumab for more than 4 years, anti-CCP positivity was identified as a predictor for BMD loss [33]. There is a significant in the number of females in ACPA-positive RA patients. We further reanalysed the Tables 2–4 using only female participants. The results were similar to the analysis of entire population (data not shown). We identified a novel differential effect of ACPA positivity on the IL-6 inhibitor-mediated suppression of a bone resorption marker, resulting in an increase in BMD. This finding supports the hypothesis that autoantibodies may influence bone metabolism and how bone turnover responds to anti-inflammatory agents. However, this finding should be verified in larger cohorts of patients with RA.

Another finding of our study is that patients who received a higher dose of glucocorticoid were not associated with bone loss through IL-6 inhibition. Glucocorticoid-induced osteoporosis is a well-known phenomenon, and a meta-analysis showed an association of steroids with a low BMD of the lumbar spine [34]. Studies have also indicated that a low dose of prednisolone may suppress inflammation, counteracting the harmful effects on bone metabolism [15, 33]. Furthermore, a randomized trial demonstrated that adding 10 mg of prednisolone to an MTX-based tight control strategy did not lead to bone loss in patients with early RA who received bisphosphonates [35]. In a prospective study of patients with active RA who received adalimumab for 1 year, a low dose of prednisolone (≤10 mg/day) improved the BMD of the femoral neck compared with no concomitant steroid use [36]. The results of these and our study contradict the traditional concept regarding the pernicious effects of glucocorticoid on bone metabolism. Strict control of inflammation, even with a low dose of glucocorticoid, might influence bone metabolism. Further study is needed to determine the effects of glucocorticoid on biologics-treated RA patients.

To the best of our knowledge, this study is the first to report the differential effects of ACPAs and glucocorticoids on bone metabolism after TCZ treatment. Our results also present a link between autoantibody production and how bone metabolism responds to IL-6 inhibition. However, our study has several limitations. First, the levels of the BTMs CTX and P1NP were measured only before and after 2-year TCZ treatment. Although the International Osteoporosis Foundation has recommended serum P1NP and CTX levels as reference BTMs, they are subject to change due to circadian rhythms, food intake, or even RA disease activity [37]. To avoid the potential effect of circadian rhythms, serum samples for measuring BTM levels were collected at 7:30 a.m. and 10:00 a.m. with overnight fasting. Moreover, changes in BMD concordantly support BTM results, suggesting that bone loss is arrested after TCZ treatment. Second, patients with active RA who failed to stay on TCZ for 2 years were not included in this study. Patients may discontinue TCZ due to uncontrolled inflammation or adverse events. This approach may underestimate the effect of RA disease activity or inflammatory pathways other than IL-6 on bone metabolism. However, the enrolment of participants who completed TCZ treatment for 2 years also provided a more homogeneous group in terms of patients’ characteristics. Third, a control group of similar patient profiles and disease activity is lacking. Nevertheless, a treat-to-target approach in RA management is a contemporary standard of care. The treatment strategy should be adjusted accordingly for patients with RA with moderate disease activity. It would be unethical to establish such a control group for 2 years. Finally, radiographic damage was not documented. Therefore, we cannot extrapolate our findings to local bone loss around inflamed joints. However, juxta-articular osteoporosis is beyond the scope of this study.

In conclusion, TCZ treatment for 2 years reduced the levels of bone resorption markers and increased the BMD of the femoral neck in patients with ACPA-positive RA. The net effects of a low dose of a steroid challenge the traditional concept of deleterious effects of glucocorticoids on bone. Tight control of disease activity with either IL-6 inhibition or prednisolone use may affect the long-term impact of inflammation on bone.

## Supporting information

S1 Fig Scatter plots of raw data. (A) osteocalcin, (B) P1NP, (C) CTX, (D) lumbar spine, (E) femoral neck, right, (F) femoral neck, left BMD, (G) lumbar spine, (H) femoral neck, right (I) femoral neck, left, T-score. BMD: bone mineral density; CTX: C-terminal cross-linking telopeptide of type I collagen; P1NP: N-terminal propeptide of type I collagen.
(TIF)

S1 Dataset Supporting information file of the raw dataset. (XLSX)
