# Supplementary figures and images for: Tocilizumab potentially prevents bone loss in patients with anticitrullinated protein antibody-positive rheumatoid arthritis
Source: PLoS One. 2017 Nov 20;12(11):e0188454. doi: 10.1371/journal.pone.0188454 (PMC5695761; doi:10.1371/journal.pone.0188454)

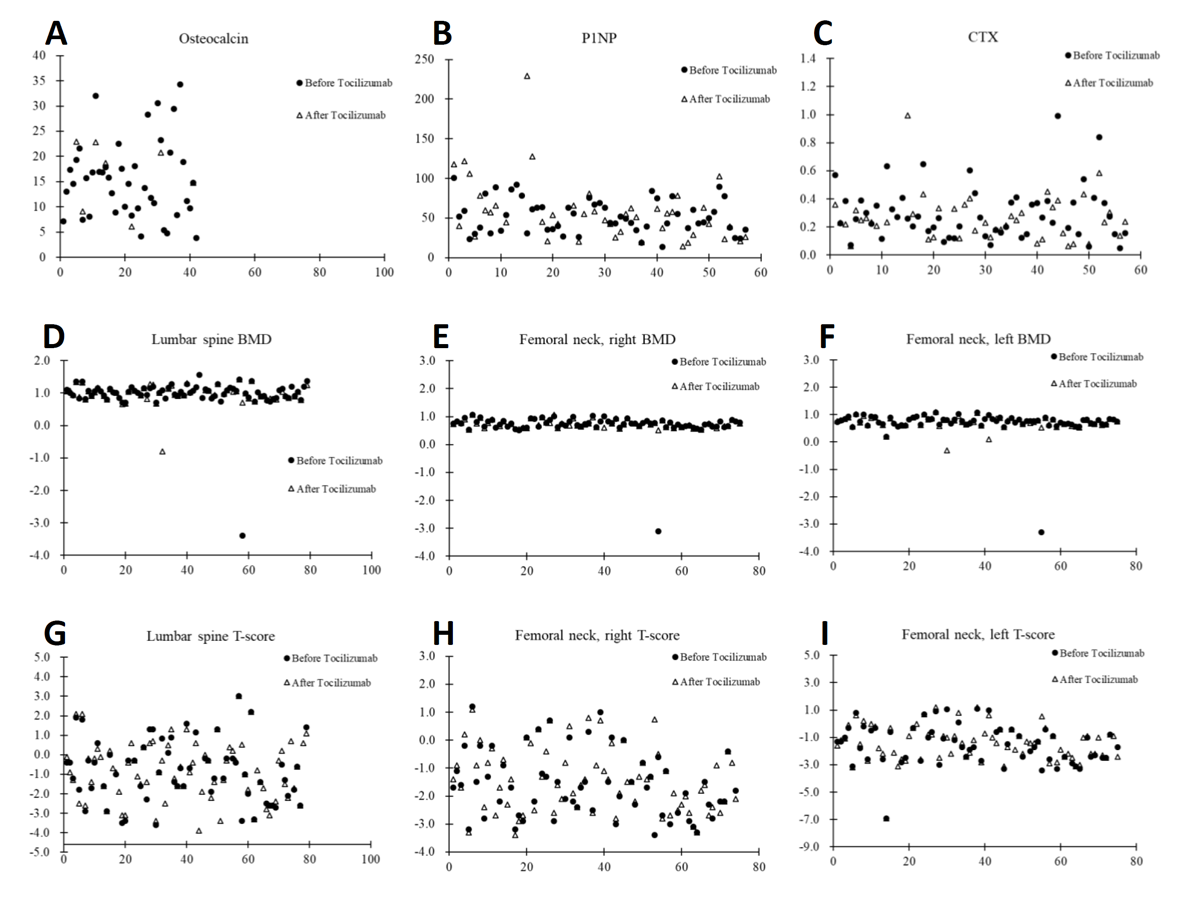

Supplement: S1 Fig — (A) osteocalcin, (B) P1NP, (C) CTX, (D) lumbar spine, (E) femoral neck, right, (F) femoral neck, left BMD, (G) lumbar spine, (H) femoral neck, right (I) femoral neck, left, T-score. BMD: bone mineral density; CTX: C-terminal cross-linking telopeptide of type I collagen; P1NP: N-terminal propeptide of type I collagen. (TIF) [file pone.0188454.s001.tif]
